# Acupoint catgut embedding for the treatment of sciatica: A protocol for a systematic review

**DOI:** 10.1097/MD.0000000000023951

**Published:** 2021-01-08

**Authors:** Yingrong Zhang, Yanji Zhang, Xia Chen, Kou Xu, Mengyuan Huang, Sanchun Tan, Zhongyu Zhou

**Affiliations:** a Hubei University of Chinese Medicine/The Co-innovation Center for Preventive Treatment of Disease of Acupuncture-moxibustion in Hubei Province; b Department of Acupuncture, Hubei Provincial Hospital of Traditional Chinese Medicine; c Hubei Province Academy of Traditional Chinese Medicine, Wuhan, China.

**Keywords:** acupoint catgut embedding, acupuncture, sciatica, study protocol, systematic review

## Abstract

**Background:**

Sciatica is a common and frequent peripheral neuropathic pain disease, which causes a great burden on peoples life. Recently, acupoint catgut embedding (ACE) has been widely applied for treating sciatica in China, however, there is no enough evidence to prove the efficiency and safety of ACE for sciatica. Our study aims to evaluate the efficiency and safety of ACE for sciatica.

**Methods and analysis:**

Searches of the Cochrane Library, PubMed, Springer Medline, EMBASE, China National Knowledge Infrastructure (CNKI), Wan-Fang Data (WANFANG), Chinese Biomedical Literature Database (CBM), and Chinese Scientific Journal Database (VIP databases) will be performed from inception to November 2020. The main outcomes are the pain intensity and the whole efficiency assessment. The secondary outcomes will include Oswestry Disability Index (ODI), life quality, physical examination, and adverse events. Two reviewers will separately conduct the study selection, data extraction and study quality assessments. RevMan 5.3 software will be used for meta-analysis

**Results:**

This study will provide an evidence-based review of acupoint catgut embedding therapy for sciatica according to the pain intensity, the whole efficiency assessment, life quality, DOI index and adverse events.

**Conclusions:**

This systematic review will present the current evidence for acupoint catgut embedding therapy for sciatica.

**Ethics and dissemination:**

Ethical approval is unnecessary as this protocol is only for systematic review and does not involve privacy data. The findings of this study will be disseminated electronically through a peer-review publication or presented at a relevant conference.

**Trial registration number:**

INPLASY2020110087.

## 1 Introduction

Sciatica is a common peripheral neuropathic pain that is mostly caused by lumbar disc herniation or aseptic inflammation. The main symptom of sciatica is low back radiating pain along the buttock, posterior aspect of thigh and leg as well the foot.^[1–3]^ Sciatica not only presents with radiating pain, paraesthesia, but have decreased muscle strength and abnormal reflexes symptoms in long run.^[4]^ In addition to physiological pain, patients could express some negative emotions including anxiety and depression. Sciatica also has a bad effect on the patients life quality and work ability.^[5]^

Two thousand sixteen 《The Lancet》 pointed out that low back pain was the main cause of global disability from 1990 to 2015, about 60% of the low back pain was sciatica.^[6]^ Clinically, 35 to 55 years old is a common population of sciatica, however, with the change of lifestyle, people always sit down whether they are working or studying. Thus the incidence of lumbar intervertebral disc prolapse is increasing, and sciatica can happen in younger patients.^[7,8]^ Suggesting that there are 5 out of every 1000 people suffering from sciatica in Western countries. In America, 25% of the medical insurance expenditure is paying for treating sciatica, and in England, the direct or indirect expenses for sciatica is £4.3 billion.^[9,10]^

Now, western medicine treatments for sciatica are surgery and conservative treatment.^[11]^ Conservative treatment mainly includes western medicine and bed rest. Western medicine can relieve pain in the short term, but there is no significant difference between western medicine and the placebo effect. What is more, the efficacy mechanism remains to be further studied, and the side effect can not be overlooked.^[12,13]^ Although surgery benefits for patients suffering from persistent sciatica, the efficiency of surgery can not last for a long time and the expenditure could not be afforded.^[14]^ Traditional Chinese medicine, especially acupuncture therapy as a safe and efficient treatment is popular among patients.^[15]^

Acupoint Catgut Embedding (ACE), a subclass of acupuncture, has rapidly developed in the past 60 years. Owning to its simple operation, obvious and sustainable efficiency, ACE is gradually applied to chronic diseases.^[16]^ ACE means to put the absorbable catgut into the acupoint. It not only remains the acupuncture effect but achieves the continuous stimulation of acupoints. ACE has been proved efficient in chronic pain, such as cervical spondylosis, lumbar disc herniation, frozen shoulder, knee arthritis in many articles.^[16–19]^ Thus ACE therapy can be further applied to pain in clinical.

Recently there are more and more articles about ACE treating sciatica.^[20]^ However, the clinical effect of sciatica with ACE remains to be seen because of its weak assessment and high risk of bias. Therefore, this review will evaluate the safety and efficiency of ACE treatment for sciatica. And our study will discuss the following questions: what is the comparative effect and safety? Does ACE therapy have an absolute advantage compared with acupuncture and western medicine? The evaluation results are intended to provide more treatment options for clinical doctors.

## 2 Methods and analysis

### 2.1 Registration

Our systematic review protocol is reported according to the Preferred Reporting Items for Systematic Reviews and Meta-Analyses Protocols (PRISMA-P) statement guidelines.^[21]^ The systematic review protocol is registered in INPLASY (2020110087;DOI: 10.37766/inplasy2020.11.0087).

### 2.2 Inclusion criteria of study selection

#### 2.2.1 Type of studies

Only the randomized controlled trials (RCTs) compared ACE therapy with other interventions conducted in human will be included, language and blinding will not be limited. Non-randomized clinical trials, observational study, case reports, animal mechanism studies will not be considered.

#### 2.2.2 Types of participants

Participants who are diagnosed with sciatica will be included and the diagnostic criteria must meet the acknowledged standards at home and abroad. There is no limit between gender and race. Patients will be excluded with low back pain caused by spinal tumors, cauda equina syndrome, and the patients with pregnancy will be absolutely excluded.

#### 2.2.3 Types of interventions

ACE as the only therapy for sciatica in clinical will be included.

Control inventions including different types of acupuncture without ACE, traditional Chinese medicine, western medicine, placebo, or sham ACE will be considered.

Invention comparing different acupoints scheme of ACE will be excluded.

The treatment group will not restrict the ACE materials, the course and frequency of treatment.

#### 2.2.4 Types of outcomes

##### 2.2.4.1 Primary outcomes

The main outcomes are the pain intensity and the whole efficiency assessment. Pain visual analogue scale (VAS) score, six-point behavior (BRS-6) score, modified Japanese Orthopaedic Association (JOA) score will be used to measure the pain intensity. The proportion of patient improvement will be used to assess the whole efficiency.

##### 2.2.4.2 Secondary outcomes

Oswestry Disability Index (ODI), life quality (EQ-5D scale, Medical Outcomes Study 36-item Short Form health survey (SF-36 scale)), physical examination and adverse events all will be taken into consideration.

### 2.3 Search methods for identification of studies

The main information source will include electronic resource databases, clinical registries. The search strategy will be conducted following the Cochrane Handbook guideline.^[22]^ The Cochrane Library, PubMed, Springer Medline, EMBASE, Web of Science, CNKI, WANFANG, CBM, and VIP databases are the main electronic resource databases used to search the key words. Clinical registries include the Chinese Clinical Trial Registry Centre (http://www.chictr.org.cn/) and the WHO International Clinical Trials Registry Platform (ICTRP) (https://clinicaltrials.gov/) searched for ongoing trials.

All the databases will be searched from the available date of inception to November 2020. Catgut implantation, catgut embedding, thread implantation, thread embedding, sciatica, bilateral sciatica is the searched key words. Search items will be the Mesh or the free words and not limited in language. And the referenced articles in the relevant studies will be searched in case of omittance. The example search strategy for the PubMed database is displayed in Table 1.

**Table 1:** Search strategy in PubMed database.

Number	Search terms
#1	“Sciatica” [Mesh]
#2	“Sciatic Neuralgia” [Mesh]
#3	Sciatica [Title/Abstract] OR Sciatic Neuralgia [Title/Abstract] OR bilateral sciatica [Title/Abstract] OR bilateral sciatics [Title/Abstract] OR Sciatic Neuralgias [Title/Abstract]
#4	#1 OR #2 OR #3
#5	thread implantation [Title/Abstract] OR thread embedding [Title/Abstract] OR acupoints catgut embedding [Title/Abstract] OR catgut implantation[Title/Abstract]
#6	“Randomized Controlled Trials as Topic” [Mesh]
#7	“Randomized Controlled Trial” [Publication Type]
#8	“Pragmatic Clinical Trial” [Publication Type]
#9	“Pragmatic Clinical Trials as Topic” [Mesh]
#10	“Intention to Treat Analysis” [Mesh]
#11	“random allocation” [Mesh Terms]
#12	random^∗^ [Title/Abstract]
#13	#6 OR #7 OR #8 OR #9 OR #10 OR #11 OR #12
#14	#4 AND #5 AND #13

### 2.4 Date collection and analysis

#### 2.4.1 Selection of studies

**Figure 1:**
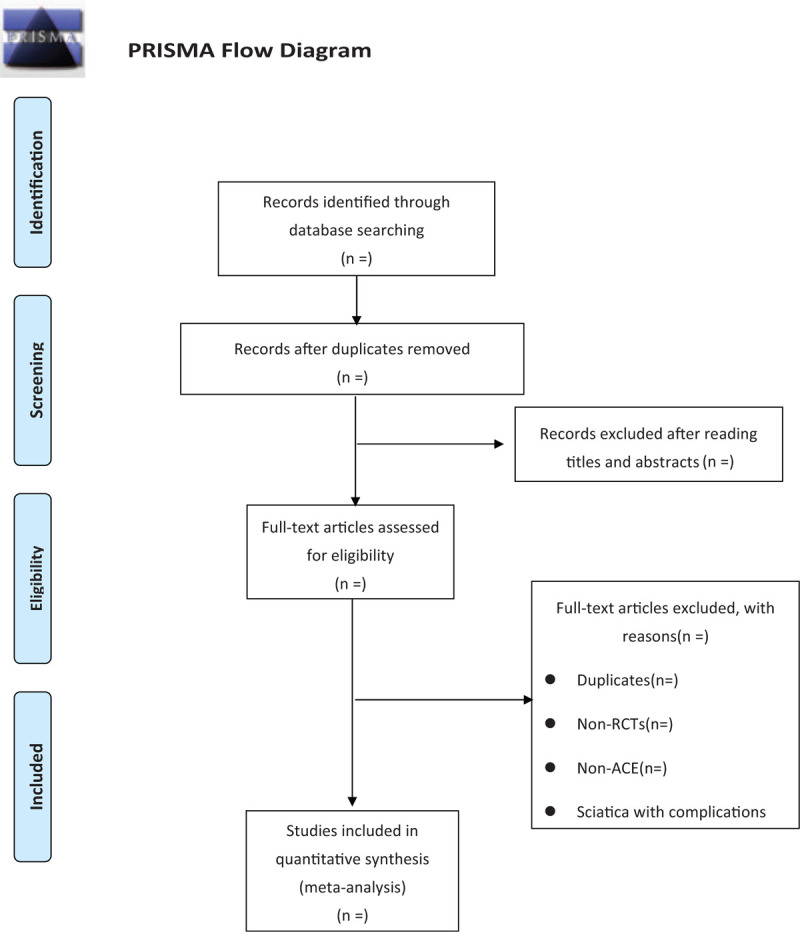
Prisma fow chart.

All the viewers will be trained to know the aim and process of the study. Two reviewers (XC and YZ) will screen the literature, extract the data, assess the risk of bias, and finally check them crosswise. First, we will read the title of the article to select the literature. Second, we will further read the abstracts and full texts to determined inclusion when excluding the non-relevant literature. The original publications will be selected to prevent the occurrence of duplicate publications. Another researcher (ST) will assist in the evaluation when disagreement occurrence. The specific selection process is shown in the PRISMA flow chart (Fig. 1).

#### 2.4.2 Data extraction and management

Two reviewers (YZ and MH) will extract and manage the dates separately. We will adopt a standard data extraction form to record the relevant informed:

1. Name of author, contact information, time of publication and country;
2. Patient characteristics, criteria of the diagnosis and improvement about sciatica;
3. Intervention details include the time, frequency and course of ACE, period of follow-up, experimental intervention details and control interventions; the revised Standard for Reporting Interventions in Clinical Trials of Acupuncture (STRICTA)^[23]^ will be used in conjunction with CONSORT^[24]^ to extract the details;
4. Risk of bias assessment, blind method, randomization method and adverse effects.

#### 2.4.3 Assessment of risk of bias in included studies

The assessment of the risk of bias in the included studies will use the Cochrane Collaboration Risk of Bias Tool,^[25]^ which will be conducted by 2 reviewers (ZZ and YZ) and another reviewer (KX) will assist in resolving the disagreements. There are 7 aspects used to evaluate the risk of bias: random sequence generation, allocation concealment, the blinding method for participants and personnel, blinding of outcome assessment, incomplete outcome data, selective reporting and other bias.

#### 2.4.4 Measures of treatment effect

Statistical analysis will be implemented based on the RevMan software (version 5.3 for Windows; the Nordic Cochrane Centre, Copenhagen, Denmark). The index of dichotomous data will be the relative risk (RR) calculated with 95% CI. For continuous variables, the mean difference (MD) will be calculated with 95% CI.

#### 2.4.5 Missing data

The respective corresponding author will be contacted to try the best to obtain any missing date. If the missing date is impossible to be obtained, we will finally exclude the study.

#### 2.4.6 Assessment of heterogeneity

Before combining the statistics, the heterogeneity will be determined based on the χ^2^ test (test level α=0.1) and the *I*^2^ statistic.^[26]^ According to the *I*^2^ statistic, we can choose the fixed effect model or random effects model. When there is an obvious clinical heterogeneity, conducting subgroup analysis or sensitivity analysis or conducting descriptive analysis will be used after assessing the reason.^[27]^

#### 2.4.7 Assessment of reporting biases

A funnel plot will be used to estimate the publication bias when more than 10 trials are included in a meta-analysis.^[28]^

#### 2.4.8 Data synthesis

RevMan (V.5.3) will be used to calculate the RR with 95% CI for dichotomous data and the MD for continuous variables. If *I*^2^ ≤ 50%, a fixed effect model will be used for the meta-analysis. If *I*^2^ > 50%, a meta-analysis will be performed using a random effects model after further analysis of the heterogeneity of the sources. Text will be provided to summarize the findings of the included publications when the data are not suitable to quantitatively combine. If trials reporting only has pre- and post-intervention values, the mean changes will be obtained according to subtracting the pre-measurements from the post-measurements. Respectively, the standard deviation (s.d.) for changes will be estimated.^[29]^

#### 2.4.9 Subgroup analysis

The type of control group and the frequency of treatment will be the basis of the subgroup.

#### 2.4.10 Sensitivity analysis

Only the test for heterogeneity of p value is less than 0.1 after subgroup analysis, the sensitivity analysis will be conducted. The low quality studies will be excluded, and the meta-analysis will be performed again.

#### 2.4.11 Quality of evidence

The quality of evidence will be reviewed according to the Grading of Recommendations, Assessment, Development, and Evaluation (GRADE). There are 4 levels to assess the quality of evidence: very low, low, moderate, or high.

## 3 Discussion

Sciatica is a clinical frequent disease and common disease,^[30]^ A lot of Meta analysis has proven that acupuncture is an effective and safe method to treat sciatica.^[11,15,30]^ ACE, as a subtype of acupuncture, has been applied to treat sciatica in China, but the efficacy and safety of it remains to be seen. At present, there is no meta-analysis about acupoint catgut embedding treating sciatica. This study will be the first meta-analysis to evaluate the efficacy and safety of ACE treating sciatica. Sciatica accounts for a large proportion of medical expenses and causes a significant burden on individual and social medical expenses.^[9,10]^ It has been proved by the clinical study that the treatment costs of acupoint catgut embedding are more affordable than those of electro acupuncture.^[31]^ When selecting the treatment, the patients economic situation should be taken into consideration in addition to effect. In China, people with sciatica should get acupuncture treatment every other day or at least 2 to 3 times a week, however, the patient is difficult to persist duo to the life or work reason, thus it is hard to guarantee the effect. Catgut embedding at acupoints uses the absorbable chorda chirurgicalis in acupoints to stimulate acupoints continuously, and the effect of ACE can persist 2 to 4 weeks,^[32]^ which greatly improves the patient compliance.

This protocol for a systematic review aims to evaluate the efficiency and the adverse events of catgut embedding at acupoints treatment for sciatica, which could provide some options for clinical treatment. Nevertheless, this study also has some limitations. Firstly, it is hard to unify the acupoints in our included clinical trials, and the course of treatment is different. Second, because of the language limitation, our study only includes the Chinese and English articles, the other language articles are overlooked.

## Author contributions

Yingrong Zhang, Yanji Zhang, and Xia Chen have contributed equally to this work. All authors have read and approved the publication of the protocol.

**Conceptualization:** Yingrong Zhang, Yanji Zhang, Xia Chen, Zhong-Yu Zhou.

**Data curation:** Yanji Zhang, Xia Chen, Kou Xu, Mengyuan Huang, Sanchun Tan.

**Formal analysis:** Yingrong Zhang, Sanchun Tan, Zhong-Yu Zhou.

**Investigation:** Xia Chen, Sanchun Tan, Zhong-Yu Zhou.

**Methodology:** Yingrong Zhang, Yanji Zhang, Kou Xu.

**Software:** Xia Chen, Kou Xu, Mengyuan Huang.

**Supervision:** Yingrong Zhang, Zhong-Yu Zhou.

**Writing – original draft:** Yingrong Zhang, Yanji Zhang, Xia Chen.

**Writing – review & editing:** Sanchun Tan, Zhong-Yu Zhou.

## References

[1] Jensen Rikke K Kongsted A Kjaer P Diagnosis and treatment of sciatica. BMJ 2019;367:l6273. 31744805 10.1136/bmj.l6273

[2] Bart W Koesab Improving the management of sciatica. Lancet Rheumatol 2020;2:e372–3. 10.1016/S2665-9913(20)30130-2 38273601

[3] Frymoyer JW Back pain and sciatica. N Engl J Med 1988;318:291–300. 2961994 10.1056/NEJM198802043180506

[4] Vos Theo Flaxman Abraham D Naghavi M Years lived with disability (YLDs) for 1160 sequelae of 289 diseases and injuries 1990-2010: a systematic analysis for the Global Burden of Disease Study 2010. Lancet 2012;380:2163–96. 23245607 10.1016/S0140-6736(12)61729-2 PMC6350784

[5] Stirling ER Patel Mohammed S Sell Philip J Sciatica. Br J Hosp Med (Lond) 2016;77:C180–3. 27828743 10.12968/hmed.2016.77.11.C180

[6] Snidvongs S Taylor Rod S Ahmad Alia Facet-joint injections for non-specific low back pain: a feasibility RCT. Health Technol Assess 2017;21:1–30. 10.3310/hta21740 PMC5742455 29231159

[7] Xiang A Xu M Liang Yan Immediate relief of herniated lumbar disc-related sciatica by ankle acupuncture: a study protocol for a randomized controlled clinical trial. Medicine (Baltimore) 2017;96:e9191. 29390461 10.1097/MD.0000000000009191 PMC5758163

[8] Marlena K Jakub PH Wiktor B Sciatica - radiating pain affecting an increasing part of society, Journal of Education. Health and Sport 2019;9: 3Suppl: 57–66.

[9] Ostelo RW Physiotherapy management of sciatica. J Physiother 2020;66:83–8. 32291226 10.1016/j.jphys.2020.03.005

[10] Maslak Joseph P Jenkins Tyler J Weiner Joseph A Burden of Sciatica on US Medicare Recipients. J Am Acad Orthop Surg 2020;28:e433–9. 31517882 10.5435/JAAOS-D-19-00174

[11] Lewis Ruth A Williams Nefyn H Sutton Alex J Comparative clinical effectiveness of management strategies for sciatica: systematic review and network meta-analyses. Spine J 2015;15:1461–77. 24412033 10.1016/j.spinee.2013.08.049

[12] Dimitroulas T Lambe T Raphael Jon H Biologic Drugs as Analgesics for the Management of Low Back Pain and Sciatica. Pain medicine (Malden, Mass) 2019;20:1678–86. 10.1093/pm/pny214 30576566

[13] Stochkendahl MJ Kjaer P Hartvigsen J National Clinical Guidelines for non-surgical treatment of patients with recent onset low back pain or lumbar radiculopathy. Eur Spine J 2018;27:60–75. 28429142 10.1007/s00586-017-5099-2

[14] Oosterhuis T Smaardijk Veerle R Kuijer P Systematic review of prognostic factors for work participation in patients with sciatica. Occup Environ Med 2019;76:772–9. 31296665 10.1136/oemed-2019-105797 PMC6817989

[15] Huang Z Liu S Zhou J Efficacy and safety of acupuncture for chronic discogenic sciatica, a randomized controlled sham acupuncture trial. Pain Med 2019;20:2303–10. 31369674 10.1093/pm/pnz167

[16] Wu X-Y Chen G-Z Li Y-T The key issues and corresponding strategy of acupoint medicated catgut embedding therapy. Zhongguo Zhen Jiu 2019;39:81–5. 30672262 10.13703/j.0255-2930.2019.01.019

[17] Won-Suk Sung Bon-Hyuk Goo Eun-Jung Kim Efficacy and safety of thread-embedding acupuncture for lumbar herniated intervertebral disc: a systematic review and meta-analysis. Europ J Integrative Med 2020;39:101195.

[18] Lee SM Ji YS Jeon JH Effect of needle-embedding & acupuncture therapy on shoulder pain in behcet disease patient: a case report. Acupunct Res 2013;30:219–24.

[19] Purumea Jun Chang-Hyun Han Chang Sop Yang Efficacy and safety of thread embedding acupuncture on knee osteoarthritis: a randomized, controlled, pilot clinical trial. Medicine 2020;99:e21957. 32899030 10.1097/MD.0000000000021957 PMC7478827

[20] Tang Jinan Liao Linfeng Seventy-two cases of sciatica treated by catgut point-embedding therapy. J Tradit Chin Med 2007;27:28–30. 17393621

[21] Kim JI Kim YI Kim E Effectiveness and safety of polydioxanone thread embedding acupuncture compared to physical therapy in the treatment of patients with non-specific chronic neck pain: study protocol for an assessor-blinded, randomized, controlled, clinical trial. Medicine 2019;98:e16768. 31393397 10.1097/MD.0000000000016768 PMC6709075

[22] Shamseer L Moher D Clarke M Preferred reporting items for systematic review and meta-analysis protocols (PRISMA-P) 2015: elaboration and explanation. BMJ 2015;349:g7647. 10.1136/bmj.g7647 25555855

[23] Higgins JP Green S Cochrane handbook for systematic reviews of interventions, version 5.1.0. Updated March 2011. Cochrane Collaboration 2011.

[24] MacPherson H Altman DG Hammerschlag R Revised STandards for Reporting Interventions in Clinical Trials of Acupuncture (STRICTA): extending the CONSORT statement PLoS Med;7 10.1371/journal.pmed.1000261 PMC2882429 20543992

[25] Boutron I Moher D Altman DG Extending the CONSORT statement to randomized trials of nonpharmacologic treatment: explanation and elaboration. Ann Intern Med 2008;148:295–309. 18283207 10.7326/0003-4819-148-4-200802190-00008

[26] Higgins JPT Altman DG Gotzsche PC The Cochrane Collaboration's tool for assessing risk of bias in randomised trials. BMJ 2011;343:d5928. 22008217 10.1136/bmj.d5928 PMC3196245

[27] Higgins JP Thompson SG Quantifying heterogeneity in a meta-analysis. Stat Med 2002;21:1539–58. 12111919 10.1002/sim.1186

[28] Lim SM Yoo J Lee E Acupuncture for spasticity after stroke: a systematic review and meta-analysis of randomized controlled trials. Evid Based Complement Alternat Med 2015;2015:870398. 25628750 10.1155/2015/870398 PMC4299539

[29] Sterne JAC Egger M Moher D Addressing reporting biases. In: Higgins JPT, Green S, eds. Cochrane Handbook for Systematic Reviews of Interventions Version 5.1.0. The Cochrane Collaboration 2011. http://www.cochrane-handbook.org.

[30] Kim K-W Park K Park H-J Effect and neurophysiological mechanism of acupuncture in patients with chronic sciatica: protocol for a randomized, patient-assessor blind, sham-controlled clinical trial. Trials 2019;20:56. 30651139 10.1186/s13063-018-3164-8 PMC6335765

[31] Huang LC Pan WY Comparation of effect and cost-benefit analysis between acupoint catgut-embedding and electroacupuncture on simple obesity. Zhongguo Zhen Jiu 2011;31:883–6. 22043672

[32] Zhang X Jia C Wang J Systematic review on the effectiveness of embedding catgut therapy for simple obesity. World J Acupunct_Moxibustion 2013;23:53–8.

